# Chitosan, Polyethylene Glycol and Polyvinyl Alcohol Modified MgFe_2_O_4_ Ferrite Magnetic Nanoparticles in Doxorubicin Delivery: A Comparative Study In Vitro

**DOI:** 10.3390/molecules26133893

**Published:** 2021-06-25

**Authors:** Deevak Ramnandan, Seipati Mokhosi, Aliscia Daniels, Moganavelli Singh

**Affiliations:** Nano-Gene and Drug Delivery Group, Discipline of Biochemistry, University of KwaZulu-Natal, Private Bag X54001, Durban 4000, South Africa; deevaksyn@gmail.com (D.R.); MOKHOSIS@ukzn.ac.za (S.M.); DanielsA@ukzn.ac.za (A.D.)

**Keywords:** magnetic nanoparticles, doxorubicin, chitosan, polyethylene glycol, polyvinyl alcohol, drug delivery, anticancer

## Abstract

Cancer-based magnetic theranostics has gained significant interest in recent years and can contribute as an influential archetype in the effective treatment of cancer. Owing to their excellent biocompatibility, minute sizes and reactive functional surface groups, magnetic nanoparticles (MNPs) are being explored as potential drug delivery systems. In this study, MgFe_2_O_4_ ferrite MNPs were evaluated for their potential to augment the delivery of the anticancer drug doxorubicin (DOX). These MNPs were successfully synthesized by the glycol-thermal method and functionalized with the polymers; chitosan (CHI), polyvinyl alcohol (PVA) and polyethylene glycol (PEG), respectively, as confirmed by Fourier transform infrared (FTIR) spectroscopy. X-ray diffraction (XRD) confirmed the formation of the single-phase cubic spinel structures while vibrating sample magnetometer (VSM) analysis confirmed the superparamagnetic properties of all MNPs. Transmission electron microscopy (TEM) and nanoparticle tracking analysis (NTA) revealed small, compact structures with good colloidal stability. CHI-MNPs had the highest DOX encapsulation (84.28%), with the PVA-MNPs recording the lowest encapsulation efficiency (59.49%). The 3-(4,5-dimethylthiazol-2-yl)-2,5 diphenyl tetrazolium bromide (MTT) cytotoxicity assays conducted in the human embryonic kidney (HEK293), colorectal adenocarcinoma (Caco-2), and breast adenocarcinoma (SKBR-3) cell lines showed that all the drug-free polymerized MNPs promoted cell survival, while the DOX loaded MNPs significantly reduced cell viability in a dose-dependent manner. The DOX-CHI-MNPs possessed superior anticancer activity (<40% cell viability), with approximately 85.86% of the drug released after 72 h in a pH-responsive manner. These MNPs have shown good potential in enhancing drug delivery, thus warranting further optimizations and investigations.

## 1. Introduction

Developing nanoscale materials as drug delivery vehicles can enhance conventional therapeutic approaches to deliver the required doses of chemotherapeutic agents safely and efficiently in cancer therapy. Most chemotherapeutic agents are administered intravenously and accumulate in tumors due to their leaky vasculature. Owing to their lack of specificity, healthy tissue is often adversely affected [1,2]. Hence, it has become essential to optimize drug delivery vehicles to target the desired cancer site, reducing side effects and poorly administered dosages [3]. Ideally, drug delivery systems (DDS) should possess cell-specific targeting, prolonged blood circulation and the response to local stimuli at pathological sites such as variations in pH, external magnetic fields and heat [2]. Researchers have risen to this challenge, and various strategies are being explored in the hope of improving therapeutic indices.

DOX has been widely employed as an anticancer drug in treating several malignancies such as leukemia, prostate, ovarian, and brain cancer, as well as the advanced stages of breast cancer [4,5]. There are two mechanisms through which DOX functions in cancer cells; (a) the intercalation of DOX into DNA affecting topoisomerase-Ⅱ-mediated DNA repair and (b) the production of free radicals that damage the cell membrane, DNA and proteins [6]. The clinical application of DOX is still limited due to its detrimental side-effects such as gastrointestinal toxicity, myelosuppression and cardiotoxicity. Nanoparticle-based drug delivery systems present inspiring methods to overcome these side effects and decrease DOX cytotoxicity [7].

Nanotechnology is a dynamic platform for the progression of effective targeted therapeutics to produce desired responses. Nanoparticles (NPs) are significant tools for varied biomedical applications owing to their small size and intrinsic characteristics [8,9]. From the many inorganic NPs explored are magnetic NPs (MNPs), which are multifunctional platforms that have attracted substantial interest for biomedical applications such as magnetic hyperthermia, magnetic resonance imaging (MRI), contrast enhancements and drug delivery systems [10,11]. MNPs can be sub-segmented into magnetic nanocomposites, pure metals and iron oxides. Iron oxides are fundamentally comprised of maghemites (γ-Fe_2_O_3_), magnetites (Fe_3_O_4_) and ferrites (MgFe_2_O_4_). These iron oxide MNPs are extensively researched for biomedical purposes as they possess favorably low cytotoxic profiles [12,13]. This can be attributed to their biodegradability, exceptional chemical stability, high magnetic susceptibility, high saturation magnetization, intrinsic biocompatibility, low sensitivity to oxidation and reactive surfaces, non-carcinogenicity, and relatively simple synthetic and functionalization procedures [14,15]. Among the many types of MNPs, superparamagnetic iron oxide NPs (SPIONs) have been the most studied, due to their idiosyncratic properties and wide range of biomedical applications [16]. Superparamagnetism is defined as the ability of MNPs to portray a strong paramagnetic environment and saturation magnetization under the influence of an external magnetic field. Additionally, they need to have the ability to lose magnetism following the removal of the external magnetic field, resulting in zero coercivity [17].

Although MNPs possess several advantages, there are shortcomings to the extensive use of MNPs as drug delivery vehicles [18]. The characteristically large surface-to-volume ratio and van der Waals forces present result in opsonization, triggering the aggregation of the MNPs and producing clusters with relatively lower magnetization profiles. It has been reported that bare MNPs are rapidly removed from the blood circulation before reaching the desired targeted site by the reticular endothelial system (RES) and confining them primarily in the liver [19]. To overcome this, the surface of MNPs must be functionalized with biodegradable and biocompatible polymers. This increases the efficiency of the delivery vehicle as they possess longer retention times in circulation [20,21]. The biocompatibility of surface-functionalization, in addition to stabilizing the MNPs, provides an available surface for the conjugation of different molecules via advanced bioconjugation chemistry. Due to these advantageous properties, biodegradable polymeric MNPs are the ideal choices as delivery vehicles for anticancer drugs. They increase the availability of the drug in tumor tissues, thereby sustaining the effect of the drug for an extended period.

Chitosan (CHI), polyvinyl alcohol (PVA) and polyethylene glycol (PEG) were the three organic polymers used in this study and have been reported previously for coating of Mg_0.5_Co_0.5_Fe_2_O_4_ nano-ferrites [21]. CHI is derived from chitin and is a biocompatible, biodegradable linear polysaccharide that contains a variety of reactive functional groups such as amines that can aid in securing the conjugation of therapeutics, imaging agents and targeting ligands [22]. PVA is a water-soluble hydrophilic organic polymer with exceptional functionalization capabilities, adhesive attributes accompanied by excellent biocompatibility and biodegradability. PVA invokes particle monodispersity and inhibits particle coagulation [23,24]. PEG is a biocompatible polymer as it bears no potential toxic functional groups. The hydroxyl functional groups at the end of the chain enable the addition of antibodies and other agents. PEG is widely employed as it can increase the circulation half-life and the cellular uptake of SPIONs [25].

Despite several attempts in the preparation of MNPs for biomedical applications, there is a scarcity in the number of MNPs employed in clinical trials. FDA-approved MNPs are employed primarily for the treatment of anemia as MRI contrast agents [26]. Hence, this study focused on the use of superparamagnetic MgFe2O4 ferrite MNPs pre-pared via the glycol-thermal procedure and functionalization of their surfaces with the biocompatible polymers CHI, PVA and PEG, respectively. Their efficient encapsulation of the anti-neoplastic drug DOX and their drug delivery capacity was evaluated in vitro with future in vivo applications in mind

## 2. Results

### 2.1. Characterization of MNPs

**Figure 1:**
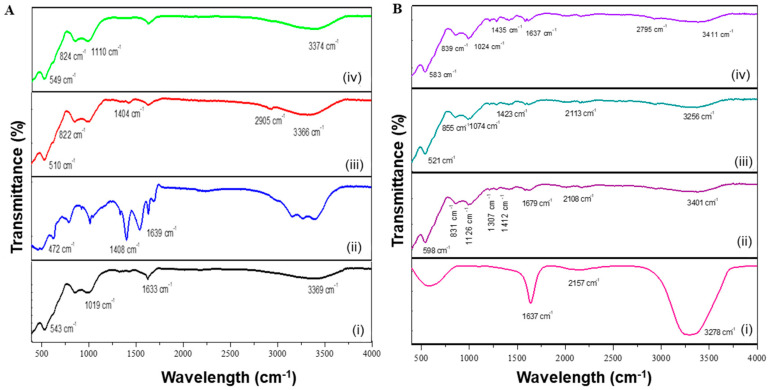
FTIR spectra for (**A**) MNPs and (**B**) DOX-loaded MNPs. **A**: (**i**) MgFe_2_O_4_, (**ii**) CHI- MgFe_2_O_4_, (**iii**) PVA- MgFe_2_O_4_ and (**iv**) PEG- MgFe_2_O_4_.; and **B**: (**i**) DOX, (**ii**) DOX-CHI-MgFe_2_O_4_, (**iii**) DOX- PVA-MgFe_2_O_4_ and (**iv**) DOX- PEG-MgFe_2_O_4_.

The composition of the synthesized MNPs and DOX encapsulation was confirmed by FTIR (Figure 1 and Supplementary Figure S1).

The functionalized MNPs possessed spectra similar to that of the uncoated MgFe_2_O_4_ MNPs indicating that functionalization did not significantly alter their composition. The encapsulation of DOX to the MNPs resulted in a slight shift of the absorption bands on the MNP spectra. The uncoated MgFe_2_O_4_ MNPs (Figure 1A-i) had an absorption peak at 3369 cm^−1^ (O-H stretching). The absorption bands situated at 1633 cm^−1^ and 1019 cm^−1^ indicated the bending mode of O-H bonds, stipulating the presence of water adsorbed on the surface of the uncoated MNPs [27]. The intense peak located at 543 cm^−1^ correlated to MTh-O-MOh stretching vibrations, with MTh being the tetrahedral and MOh the octahedral positions occupied by the MNP. This indicated that MgFe_2_O_4_ was a spinel ferrite [28]. The absorption peaks located at 1408 cm^−1^ and 1639 cm^−1^ for the CHI-MgFe_2_O_4_ (Figure 1A-ii) result from vibrations obtained from the CH_3_ functional groups and N-H bending, respectively. PVA-MgFe_2_O_4_ MNPs (Figure 1A-iii) has an absorption band present at 3366 cm^−1^ (O-H stretching) with a minor peak at 2905 cm^−1^ (C-H stretching vibrations). Additionally, the absorption bands detected at 1404 cm^−1^ and 822 cm^−1^ correlated to stretching vibrations of C-C bonds and CH_2_ rocking, respectively [28].

The spectrum of PEG-MgFe_2_O_4_ MNPs (Figure 1A-iv) showed an absorption band at 3374 cm^−1^ which correlated to intramolecular stretching vibrations of hydrogen bonds, with a characteristic absorption peak at 1100 cm^−1^ due to the repeated –OCH_2_CH_2_- groups of the PEG_2000_ backbone and correlated to the stretching vibrations of the C-O-C bonds. The absorption band located at 824 cm^−1^ results from the out of plane bending vibrations of the C-H bonds [12,29]. Furthermore, the peaks detected at 472 cm^−1^, 510 cm^−1^, and 549 cm^−1^ for Figure 1A-ii−iv infer that the polymers did not alter the spinel cubic structures following functionalization. The FTIR spectra obtained for DOX (Figure 1B-i) depicts several absorption bands at 3278 cm^−1^ (O-H), 2157 cm^−1^ (C-N) and 1637 cm^−1^ (N-H) [30]. The vibrational frequencies mentioned for the functional groups stated for the MNPs (Figure 1A) are still present for the DOX-loaded MNPs. However, the conjugation of DOX onto the functionalized MNPs induced a shift in the vibrational frequencies (Figure 1B-ii−iv).

**Figure 2:**
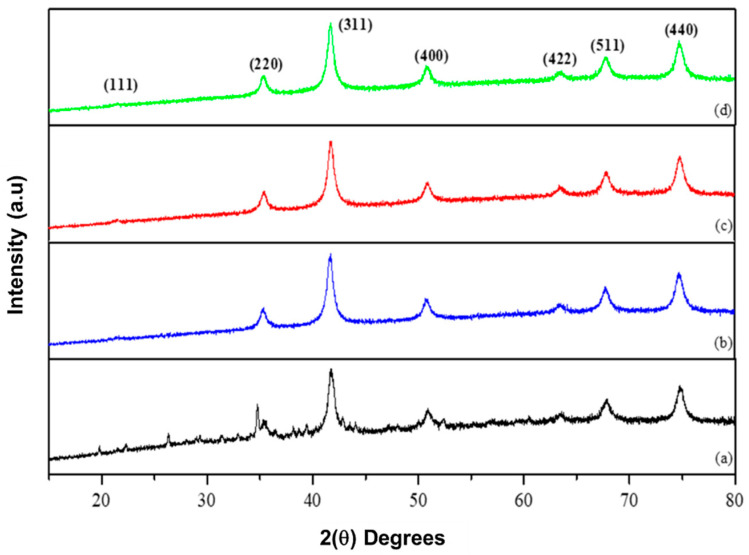
XRD patterns obtained for (**a**) MgFe_2_O_4_, (**b**) CHI-MgFe_2_O_4_, (**c**) PVA-MgFe_2_O_4_ and (**d**) PEG-MgFe_2_O_4_ MNPs.

The Joint Committee on Powder Diffraction Standards (JCPDS) spinel indexing card numbers (77-0426) and (80-0072) was used to confirm the diffraction peaks acquired from XRD (Figure 2) with the structural parameters of the MNPs obtained from XRD measurements presented in Table 1.

The diffraction peaks observed at (111), (220), (311), (400), (422), and (511) and (440) for MgFe_2_O_4_ correspond to the reflections of high intensity at 2θ values (Figure 2). These diffraction peaks are coherent with the JCPDS spinel indexing card and confirm that MgFe_2_O_4_ is a single-phase cubic spinel structure [31]. The diffraction peaks stated above are still present for the functionalized MNPs and indicate that the functionalized MNPs retained the single-phase cubic spinel structure after coating, and further signifies the stability of the crystalline phase of the MNPs following polymer functionalization [32]. XRD analysis revealed that the addition of the polymers to the surface of the MNPs resulted in the suppression of specific diffraction peaks, which can be attributed to lattice strain between the surface of the MNPs and the polymer, which emanates in the decrease of the diffraction peaks intensity [27,33]. All diffraction peaks observed do not depict any secondary phase signatures, confirming the purity of the single-phase cubic spinel structures [34]. Using Scherrer’s equation, it was deduced that functionalization of MgFe_2_O_4_ increased the average crystalline sizes of the MNPs with a decrease in lattice strain (Table 1). Decreasing lattice strain can be accounted for by the decrease of dislocations, long-range interval stress, coherency strains and crystallite largeness relative to the cubic spinel structure [35]. The lattice parameters obtained for the MNPs correlate to that in literature [36].

**Figure 3:**
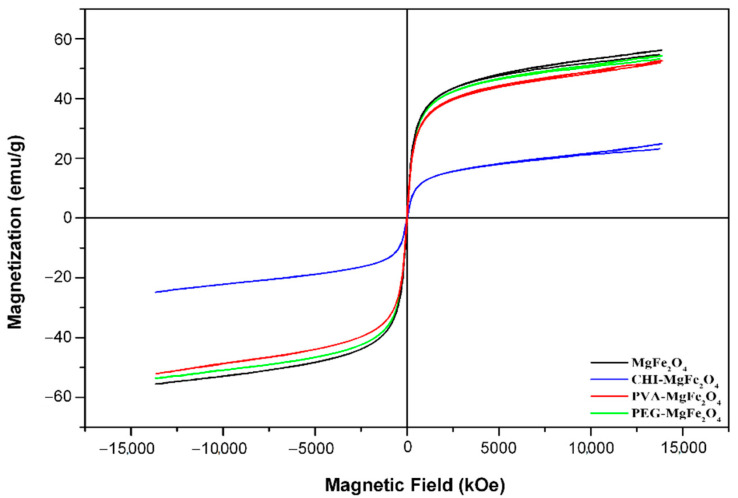
VSM magnetization profile of the MgFe_2_O_4_, CHI-MgFe_2_O_4_, PVA-MgFe_2_O_4_, and PEG-MgFe_2_O_4_ MNPs.

VSM analysis revealed hysteresis loops (Figure 3) for all the MNPs, confirming their superparamagnetic properties [37]. This can be additionally accredited to the minute sizes <25 nm of the MNPs as seen for XRD (Table 1) [38]. The observed superparamagnetic behaviour of the MNPs remained unchanged after functionalization of MgFe_2_O_4_, as evidenced by the presence of the “S” shape of the hysteresis loops in conjunction with adequate saturation magnetization values (Ms) and relatively negligible coercivities (Hc) (Figure 3 and Table 2). The Ms values from VSM analysis of the MNPs were between 24.877–55.900 emu/g (Table 2). Previous studies implied that, with polymer functionalized ferrites, the coated layer is considered to be a “dead” layer at the surface of the MNP, and reduction in saturation magnetization would consequently be a result of the quenching of surface moments [39]. The results, therefore, suggest that functionalization resulted in marginal shielding of saturation magnetization values. Coercivity is associated with the intensity of an applied external magnetic field required to reduce the magnetization of an object to zero [40]. The coercivities for the MNPs were between 3.24–8.48 KOe (Table 2).

It was observed that coercivity increased with a decrease in saturation magnetization values. The increase in the functionalized MNPs’ coercivity was due to the increase of the interparticle distance between the magnetic core of the iron oxides due to the non-magnetic effect of CHI, PVA, and PEG. This results in a weak coupling of the magnetic dipole moments. The magnetic dipole moments of the uncoated MgFe_2_O_4_ were more efficiently coupled, and therefore, a lower coercivity was reported for MgFe_2_O_4_ (Table 2) [37]. The coercivities in conjunction with the saturation magnetization values validate the superparamagnetic behaviour of the MNPs and confirm that the synthesized ferrite MNPs are SPIONs.

### 2.2. Encapsulation Efficiency

The encapsulation efficiency (%) of DOX was quantified as 84.28% (1.69 mg), 51.49% (1.03 mg) and 79.38% (1.59 mg) for CHI-MgFe_2_O_4_, PVA-MgFe_2_O_4_, and PEG-MgFe_2_O_4_ MNPs, respectively. The nanocomplexes were examined again after 6 months to determine and loss of the encapsulated drug over this time again after 6 months and shown to be close to the original encapsulation efficiencies (83.1%, 51.2% and 78.81%, respectively). This suggested that very little if any of the drug had leached out of the nanocomplex.

### 2.3. TEM and NTA Studies

**Figure 4:**
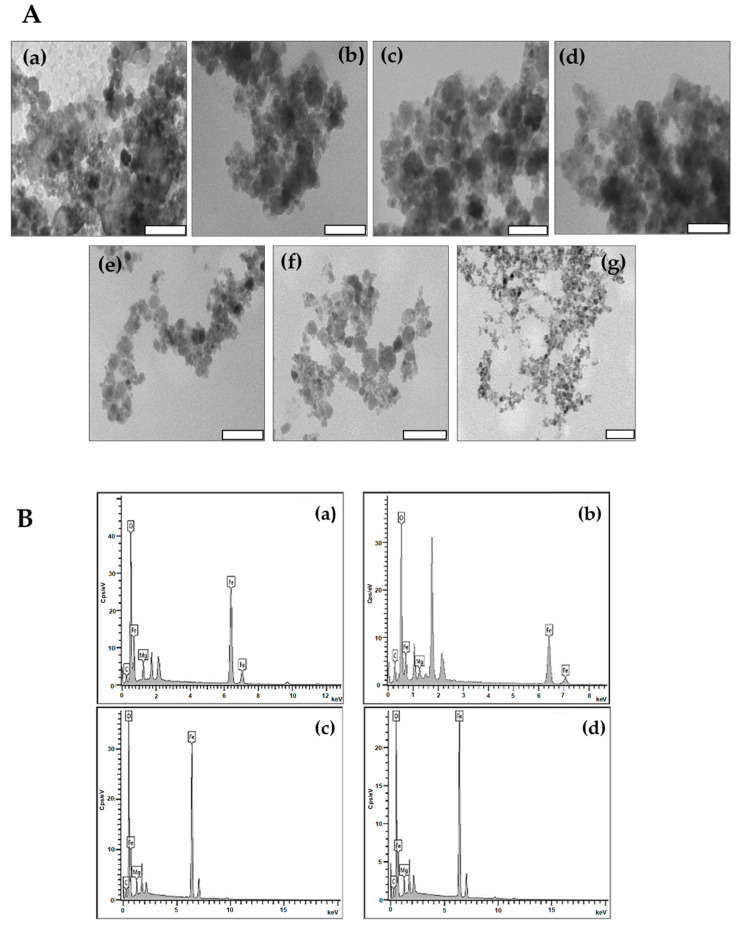
(**A**) TEM micrographs of (**a**) MgFe_2_O_4_, (**b**) CHI-MgFe_2_O_4_, (**c**) PVA-MgFe_2_O^4^, (**d**) PEG-MgFe_2_O_4_, (**e**) DOX-CHI- MgFe_2_O_4_, (**f**) DOX-PVA-MgFe_2_O_4_ and (**g**) DOX-PEG-MgFe_2_O_4_. Scale Bar = 100 nm (a-f) and 200 nm (g). (**B**) EDX micrographs for (**a**) MgFe_2_O_4_, (**b**) CHI-MgFe_2_O_4_, (**c**) PVA-MgFe_2_O_4_ and (**d**) PEG-MgFe_2_O_4_ MNPs.

TEM micrographs (Figure 4A) of MNPs and DOX-loaded MNPs revealed NPs with a quasi-spherical morphology. Following functionalization separately with CHI, PVA and PEG, there was an increase in the dispersibility of the NPs (Figure 4Ab–d), which can be accredited to the existence of the non-magnetic polymer surface layer, decreasing the interparticle interactions [41].

The elemental composition of the MNPs was further identified by EDX as depicted in Figure 4B and tabulated in Table 3.

The percentages attained for CHI-MgFe_2_O_4_, PVA-MgFe_2_O_4_ and PEG-MgFe_2_O_4_ depict the altering of the elemental composition following functionalization with the polymers. This further resulted in the hindering of the elements percentage abundances. The percentage of abundances attained correlated with the formulae of MgFe_2_O_4_, CHI-MgFe_2_O_4_, PVA-MgFe_2_O_4_ and PEG-MgFe_2_O_4_, respectively, and confirmed the successful binding of the polymers to the surface of the MNPs.

Nanoparticle tracking analysis (NTA) provided the hydrodynamic sizes and the colloidal stability of the MNPs in an aqueous solution. The MNPs and DOX-loaded MNPs were between 78 and 140 nm in size using NTA and between 16 nm and 24 nm under TEM (Table 4). Zeta potential measurements provided an assessment of the colloidal stability of the MNPs. Zeta potential values of ±0 to 10 mV, ±10 to 20 mV, ±20 to 30 mV and above ±30 mV and are used to indicate highly unstable, relatively stable, moderately stable and extremely stable particle dispersions, respectively [42]. The zeta potentials obtained for the coated MNPs and their DOX nanocomplexes were higher than that for the uncoated MNPs, confirming improved stability due to polymer functionalization

PDI measures NP heterogeneity. Monodispersed NPs typically have PDI values < 0.1, which further indicates size uniformity [43]. The PDI values attained for the MNPs, and DOX-loaded MNPs were well below 0.1, indicating that these MNPs and their drug nanocomplexes are monodispersed with a slight tendency to agglomerate.

The physical stability of the three nanocomplexes were investigated using NTA, at varying the pH and temperature as seen in Supplementary Tables S1–S3. The DOX-CHI-MgFe_2_O_4_ and DOX-PEG-MgFe_2_O_4_ nanocomplexes did seem to increase in size at lower pH especially at pH 4.5 and at lower temperature (4 °C). This could be due to some disruption of the forces that hold the complex together resulting in a loose conformation rather than a more condensed one. The zeta potentials were all below −21 mV suggesting that there was some loss of stability of the nanocomplexes especially at low pH and temperature. This could have also further induced some aggregation of the nanocomplexes causing an increase in size. The DOX-PVA-MgFe_2_O_4_ nanocomplexes, however, did not completely follow this trend, with a decrease in size at 4 °C, suggesting a tighter complex or possibly greater leaching of the DOX resulting in a smaller nanocomplex.

### 2.4. DOX Release

**Figure 5:**
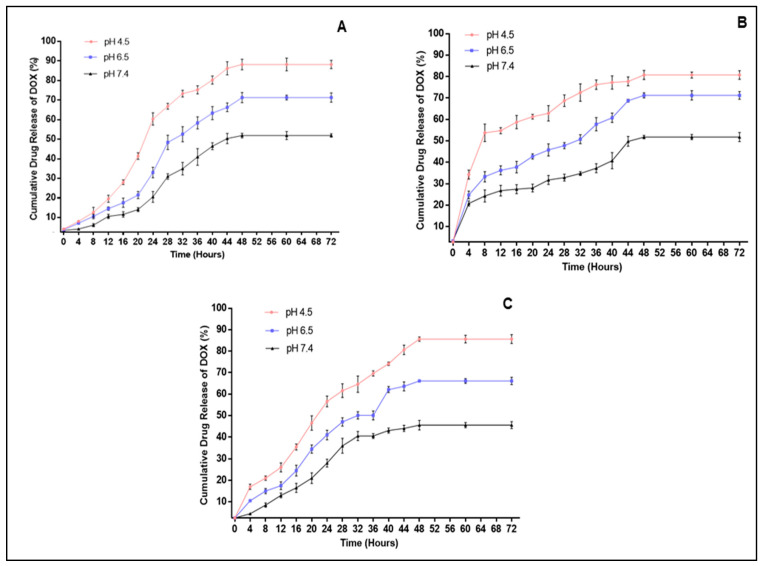
In vitro drug release profile of DOX from (**A**) DOX- CHI-MgFe_2_O_4_, (**B**) DOX-PVA-MgFe_2_O_4_ and (**C**) DOX-PEG-MgFe_2_O_4_ MNPs at pH 4.5, 6.5 and 7.4.

DOX release was measured in different pH environments, viz., pH 4.5, 6.5 and 7.4, which depicted the pH of endosomes and lysosomes, the pH of the tumor microenvironment and the pH of blood, respectively [18,44]. The in vitro drug release profiles of DOX were attained by quantifying the amount of released DOX relative to the quantity of the DOX encapsulated in the MNPs. Figure 5 confirms that the rate of DOX release was pH-dependent, increasing with a decrease in pH. Approximately 85.86%, 68.68% and 49.38% of DOX was released from the CHI-MgFe_2_O_4_ MNPs at pH 4.5, 6.5 and 7.4, respectively, after 48 h with a sustained release after 72 h. A similar trend was observed for PVA-MgFe_2_O_4_ and PEG-MgFe_2_O_4_ MNP formulations. PVA-MgFe_2_O_4_ exhibited a rapid release of DOX at 12 h at pH 4.5, and 6.5 with 32.86% and 52.08% of DOX released, respectively. This was followed by approximately 68.33% and 74.2% of DOX released after 48 h. This pH-dependent release of DOX was also evident for PEG-MgFe_2_O_4_, with the greatest amount of DOX being released at pH 4.5 after 48 h (83.93%).

### 2.5. MTT Cytotoxicity

**Figure 6:**
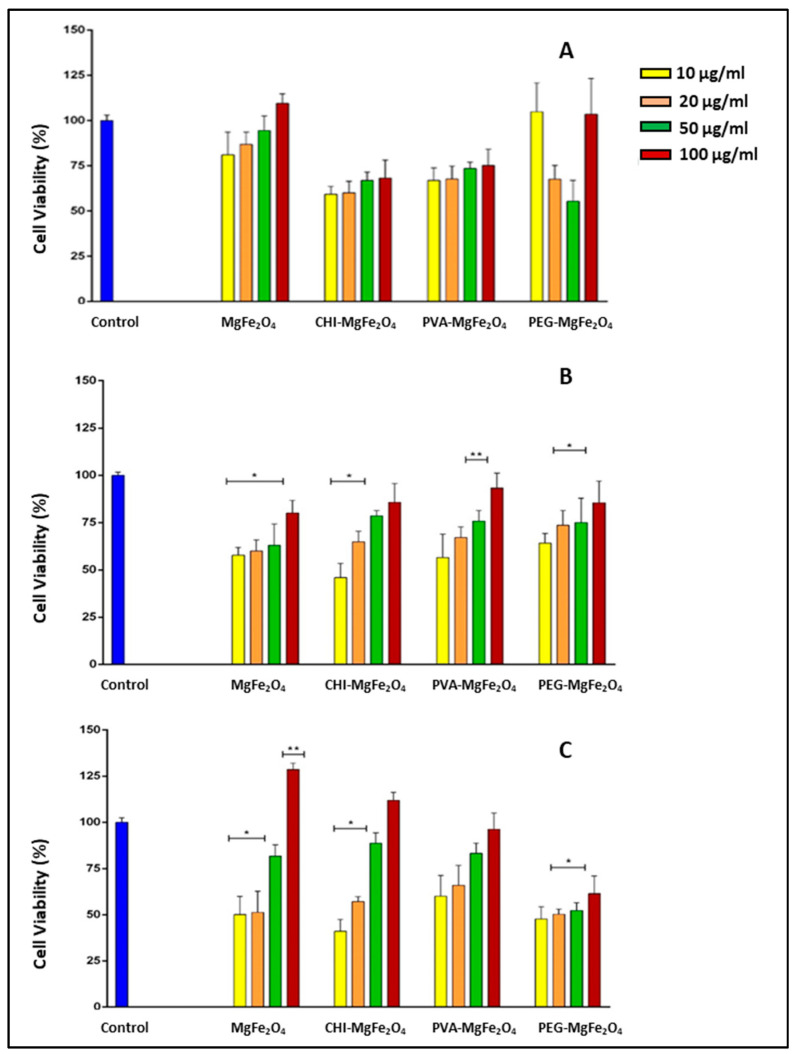
Cytotoxic profile for MgFe_2_O_4_, CHI-MgFe_2_O_4_, PVA-MgFe_2_O_4_ and PEG-MgFe_2_O_4_ MNPs in the (**A**) HEK293, (**B**) Caco-2 and (**C**) SKBR-3 cell lines, respectively. Data represented as means ± SD (*n* = 3), where * *p* < 0.05 and ** *p* < 0.01 are considered to be statistically significant.

All MNP formulations exhibited similar trends in the three cell lines tested, with a dose-dependent increase in cell viability with an increase in the concentration of the MNPs. Cells treated with the MNP formulations showed viabilities >55% in the HEK293 and Caco-2 cells with the CHI-MgFe_2_O_4_ and MgFe_2_O_4_ MNP formulations exhibiting the greatest cytotoxicity (Figure 6A,B). However, negligible toxicity was evident in SKBR-3 cells (Figure 6C). Cell proliferation was observed at 100 µg/mL with cell viabilities of 128.48% and 114.23% obtained for the MgFe_2_O_4_ and CHI-MgFe_2_O_4_ MNPs, respectively. PEG-MgFe_2_O_4_ MNPs induced slightly higher cytotoxicity than the other MNP formulations in the SKBR-3 cells, which could be attributed to the disturbance of the structure and function of the cell membranes by PEG [45].

**Figure 7:**
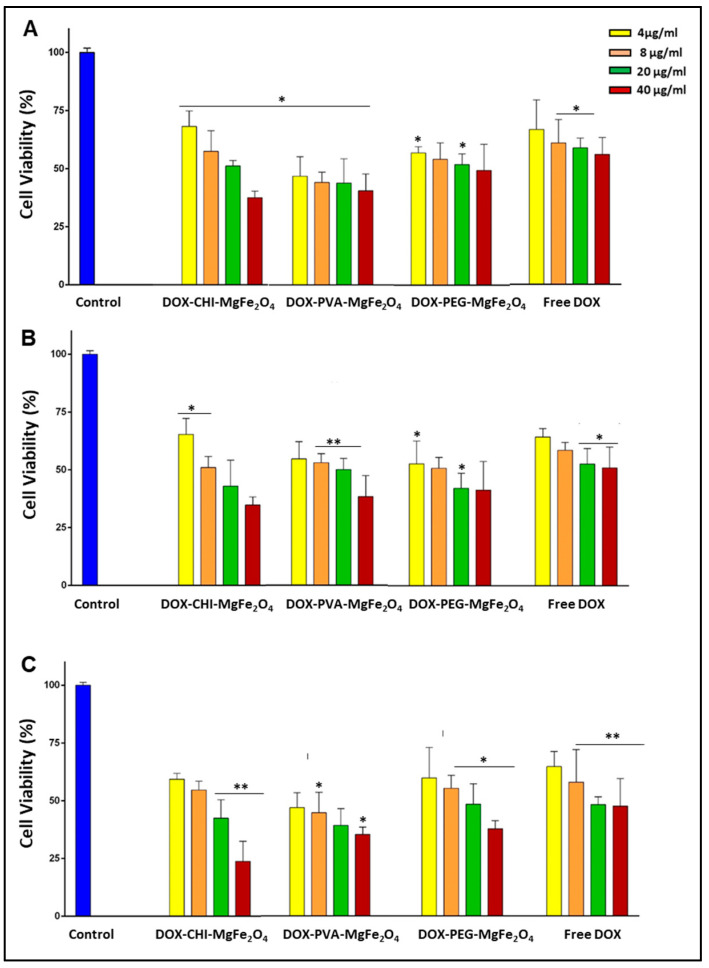
Cytotoxic profile for DOX-loaded MNPs and the free DOX in the (**A**) HEK293, (**B**) Caco-2 and (**C**) SKBR-3 cells. Data are represented as means ± SD (*n* = 3), where * *p* < 0.05 and ** *p* < 0.01 are considered statistically significant.

For the DOX-loaded MNP nanocomplexes, an increase in the concentration resulted in a decrease in cell viability in all cells, suggesting a dose-dependent cytotoxicity profile (Figure 7). The DOX-loaded MNPs presented a considerable increase in cytotoxicity at lower concentrations compared to their drug free MNP counterparts. The free DOX possessed a lower cytotoxicity compared to the DOX-loaded MNPs in the cancer cells (Caco-2 and SKBR-3). It was important to note that greater cytotoxicity was evident for the DOX-loaded MNPs in the cancer cells compared to the non-cancer HEK293 cells. The viabilities of cells treated with 20 µg/mL of DOX-CHI-MgFe_2_O_4_ MNP formulations were 42.94%, 42.49% in the Caco-2 and SKBR-3 cells, respectively, while at 40 µg/mL, the cell viabilities were 34.81% and 23.81% for the Caco-2 and SKBR-3 cells, respectively (Figure 7B, C).

Furthermore, at a concentration of 40 µg/mL, the DOX-CHI-MgFe_2_O_4_ MNP formulation had the highest anticancer activity in the cancer cells. This corroborates the results from the drug release where this nanocomplex released the most amount of DOX at the lower pH values. At 20 µg/mL DOX-PVA-MgFe_2_O_4_ MNPs possessed cell viabilities of 47.12%, 50.49%, 39.47% in the HEK293, Caco-2 and SKBR-3 cells (Figure 7), respectively. The cytotoxicity of DOX-PVA-MgFe_2_O_4_ and DOX-PEG-MgFe_2_O_4_ MNPs revealed that these nanocomplexes were also successful in DOX delivery with cell viabilities < 48% in the Caco-2 and SKBR-3 cells at the higher concentrations (20–40 µg/mL) (Figure 7). Overall, the DOX-loaded MNPs were effectively internalized with increased chemotherapeutic efficiency due to the drug being released following endocytic internalization in the cancer cells.

The IC_50_ values were calculated (Table 5 and Table 6) to determine the treatment dose required to achieve 50% of cell death [46]. Assuming DOX was the only anticancer agent active in the NP the pseudo-IC_50_ values based only on the quantity of DOX was also calculated (Table 6). Overall, the IC_50_ values for the DOX-loaded MNPs revealed that these nanocomplexes required lower doses than free DOX to achieve 50% cell death. This result confirms that the DOX-loaded MNPs have a higher anticancer activity than free DOX.

### 2.6. Apoptosis

**Figure 8:**
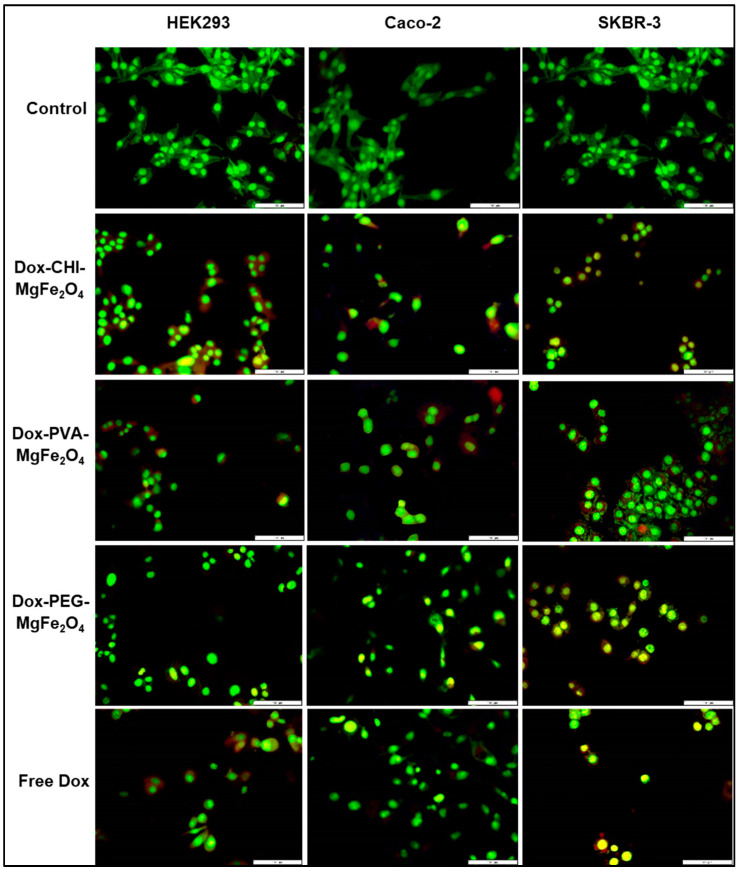
Fluorescent micrographs obtained from the dual AO/EB apoptotic study in the HEK293, Caco-2 and SKBR-3 cell lines at 100× magnification. Bar = 100 µm.

Apoptosis is a highly regulated mechanism of cell death that is categorized by a variety of biochemical alterations and distinct cellular morphology, which includes nuclear fragmentation, chromatin condensation, membrane blebbing or reduced cell volume and the formation of surface vesicles. In the apoptotic images, live cells were green, with red and orange being apoptotic and necrotic cells, respectively (Figure 8). The IC_50_ values for the DOX-loaded MNPs (Table 5) were utilized for the apoptosis evaluation. The apoptotic images reinforced the findings from the in vitro cytotoxicity studies (Figure 7), suggesting that the DOX-loaded MNPs were internalized more readily by the cancer cells viz. Caco-2 and SKBR-3. These results were verified by the apoptotic features portrayed by the DOX-loaded MNPs, including nuclear fragmentation and the increased formation of necrotic and apoptotic bodies. The apoptotic indices acquired for the DOX-loaded MNPs were higher than that for free DOX, confirming that the DOX-loaded MNPs were internalized more efficiently by the cells (Table 7).

## 3. Discussion

MNPs have attracted much attention due to their remarkable physical and chemical properties and their capacity to operate on a molecular and cellular level [47]. SPIONs intrinsically possess remnant magnetization following the removal of an external magnetic source, which will aid in minimizing or preventing coagulation, hence reducing the potential of aggregation in vivo in contrast to other MNPs [48]. Initial characterization confirmed that the MNPs utilized in this study were successfully synthesized using the glycol-thermal method, functionalized with CHI, PVA and PEG and complexed to DOX. The MNPs were small, quasi-spherical in shape, monodispersed, with a single-phase spinal structure and possessed superparamagnetic properties confirming that the prepared MNPs were SPIONS, in agreement with that reported in the literature [31]. Conjugation of DOX to the MNPs was confirmed by FTIR and TEM.

The average particle sizes from TEM were observed to be much smaller than the hydrodynamic sizes from NTA (Table 4). This could be because NTA measured particles in an aqueous medium, which could have caused swelling of the NPs, while TEM measured the NPs in their dry state [49,50]. Hence, a hydration layer may have formed around the MNPs due to the various interactions between the functional groups located on the polymer surface and the surrounding aqueous medium.

The functionalized MNPs possessed larger hydrodynamic sizes than the uncoated MgFe_2_O_4_, as reported previously [51]. The average particle size and hydrodynamic sizes attained for the DOX-loaded MNPs were comparatively smaller than their unloaded counterparts. This can be attributed to the hydrophilic groups present on the polymers of the functionalized MNP surface, which allows for efficient encapsulation of DOX through its hydrophobic cavity. Hence, interactions between the hydrophobic and hydrophilic functional groups resulted in more compact hydrophobic cores being formed, which can supplement different hydrophilic drugs into the MNP core [52,53]. NPs that are approximately 100 nm in size are considered ideal, as foreign bodies greater than 100 nm are readily removed by RES [54]. The DOX-loaded MNPs had hydrodynamic sizes below 100 nm (Table 5) and were moderately stable, suggesting that they possess the desired physicochemical characteristics for NPs in drug delivery. MgFe_2_O_4_ has a high valency (Mg^2+^) which causes compression of the electric double layer (EDL), consequently resulting in a decrease in zeta potential [55]. These results imply that the uncoated MgFe_2_O_4_ is more unstable in the aqueous solution, with functionalization increasing its stability [56]. The zeta potential attained for DOX-loaded MNPs suggests that these nanocomplexes are moderately stable, which aids in the repulsion of the NPs in the aqueous suspension, and preventing particle aggregation [57,58].

The nanocomplexes did show some change in size and stability upon variation in pH and temperature. Increases in size at low pH and low temperature could be attributed to some changes in the inter- and intra-molecular attractions within the nanocomplex resulting in looser (DOX-CHI-MgFe_2_O_4_ and DOX-PEG-MgFe_2_O_4_) or tighter structures (DOX- PVA-MgFe_2_O_4_ at 4 °C). Furthermore, lower zeta potentials could have caused aggregation of the nanocomplexes causing an increase in size. Changes in size due to change in temperature has been reported to be due to poor cross-linking of molecules at lower temperatures [59]. PVA has further been reported to possess thermal stability and chain flexibility [60] which could have contributed to the condensed DOX- PVA-MgFe_2_O_4_ nanocomplex at 4 °C and larger complexes at higher temperature. Overall, there was minimal changes in size especially at physiological pH, suggesting that the leaching of the DOX would be minimal under normal conditions, but severe changes in conditions may result in loss of conformations and leaching of the drug. Hence, the appropriate storage of such nanocomplexes do become important in these instances.

The prepared MNPs were capable of DOX encapsulation (>50%), with CHI-MgFe_2_O_4_ showing the highest DOX loading at 84.28%. PVA-MgFe_2_O_4_ had the lowest DOX encapsulation efficiency at 51.49%, which could be attributed to the low PVA concentration used for functionalization (3 wt.%). The mechanism for DOX encapsulation to PVA-MgFe_2_O_4_ involves -NH_2_ groups of DOX conjugating to the active -OH groups of PVA. The lower drug adsorption observed could be due to a decrease in PVA on the surface of the MNPs. Previous studies using CHI functionalized bimetallic NPs exhibited over 70% DOX encapsulation [61], while CHI functionalized mesoporous silica NPs presented with >90% DOX encapsulation [62]. For CHI-MgFe_2_O_4_ van der Waals interactions are known to play a significant role in DOX loading, with hydrophobic interactions also contributing. Overall, the use of CHI as a polymer seems to imbue the NPs with favorable properties for drug loading. The entrapment of DOX onto the PEG-MgFe_2_O_4_ NPs could be due to hydrogen bonding via the -NH2, -OH, C-O-C, -C and -O groups of DOX with the –OH, C-O-C, -C and -O groups on the surface of the PEG-MgFe_2_O_4_ NPs. Notably, the amount of DOX loaded into each nanocomplex showed little or no change after six months, suggesting little or no loss or leaching of DOX over time.

The in vitro drug release profile of DOX alluded to a pH-responsive nature of these MNPs, with an increase in DOX release observed in an acidic environment. All MNP formulations exhibited the highest amount of drug release at pH 4.5 and 6.5 (Figure 5). This characteristic is favorable for a drug delivery system since the pH in the tumor environment is pH 6 and below, and lower pH values (between 3 and 5.5) are found in endosomes and lysosomes in cancer cells, which suggests a favorable DOX release from the MNPs in in these microenvironments [63]. The high cumulative release of DOX at pH 4.5 can also be attributed to the increased solubility of DOX in an acidic environment [64]. Furthermore, a minimal amount of DOX was released at a physiological pH, an environment found in healthy cells. This would significantly decrease toxicity in healthy cells leading to a reduction in collateral side effects.

The examination of the cytotoxicity of a nano-delivery vehicle is crucial as the primary objective of the anti-neoplastic drug DOX is to kill cancerous cells [65]. The surface properties of both uncoated and functionalized MNPs are considered significant factors that influence cytotoxicity, with reports suggesting that the direct interaction between these MNPs and cells can be held accountable for the leaching of more iron, resulting in iron overload and subsequent cell death [66]. On a molecular level, degradation, relocation and crosslinking of proteins and DNA fragments and DNA strand breakage can occur [67,68].

Efficient uptake of DOX by cancer cells in order to allow for nuclear localization requires that the drug be released from the drug-MNP complex. This is primarily influenced by the size, composition and surface properties of the delivery vehicle [69]. The biotherapeutic properties of the DOX-loaded MNPs were investigated using the MTT cell-based assay in the HEK293, Caco-2 and SKBR-3 cells. The MTT assay examines the reduction of MTT salts that occur only in metabolically active cells. The HEK293 cell line was employed as a control non-cancer cell line to compare the cytotoxicity of the MNPs, DOX-loaded MNPs and free DOX to that in the cancer cell lines viz. Caco-2 and SKBR-3. The cytotoxicity profiles of the MNPs revealed that the CHI-MgFe_2_O_4_ MNPs showed slight toxicity to the HEK293 and Caco-2 cells. This can be attributed to CHI-MgFe_2_O_4_ possessing a positively charged amine group, which has been reported to have the potential to be more lethal due to the strong interactions of the amine groups with the negatively charged cell surface [70,71].

However, in the SKBR-3 cells, the proliferation of cells was evident at a concentration of 100 µg/mL. The proliferation of cells over 100% has been reported to be due to the ferric irons present in the ferrite MNPs reacting and enzymatically metabolizing in the cells and the cell culture medium [72]. PVA-MgFe_2_O_4_ MNPs were reasonably well tolerated by all cell lines being tested with cell viabilities greater than 60%. PEG-MgFe_2_O_4_ MNPs appeared to be more cytotoxic to the SKBR-3 cells compared to the other MNP formulations at the higher concentrations. This decrease in cell viability can be attributed to the adsorption of the PEG-MgFe_2_O_4_ on cell surfaces resulting in the disturbance of the cell membranes structure and function [45]. Furthermore, the PEG-MgFe_2_O_4_ could have induced oxidative stress in the cells. It has also been reported that incubating MNPs with cells can alter the cell surface roughness, leading to alteration of the cell’s morphology and change in the cellular cytoskeleton response resulting in cell death [73].

The proliferation noted for the three polymers functionalized MNPs as the concentration increased, could be due to their good biological properties such as biocompatibility, biodegradability, and low toxicity, which are favourable for drug delivery. The organic polymers PEG and PVA are known to improve colloidal stability by introducing steric repulsion, and limiting non-specific binding to cell receptors. It was proposed that when chitosan based nanocomplexes interact with the cell membrane, growth factors are stimulated leading to cellular proliferation. This promotion in cell growth was used as an indication that the cells had maintained their morphology and adhesion capacity [74]. A study using different molecular weights of PEG also revealed that higher molecular weights of PEG did increase cellular proliferation in colon cancer cells [75]. In the case of PVA, it was reported that due to the low protein affinities of PVA, and its limited ability to absorb to the extracellular matrix that supports cellular attachment, it does not cause any negative influences on cellular proliferation [76]. These reports support the results obtained for the functionalized MNPs in vitro.

A dose-dependent cytotoxic profile was evident for the DOX-loaded MNP formulations, with more significant cell death observed in the Caco-2 and SKBR-3 cells compared to the HEK293 cells. Overall, the DOX-loaded MNPs showed better anticancer activity than free DOX. Following the uptake of the DOX-loaded MNPs by the cancer cells, DOX is released into the nucleoplasm through the nuclear membrane, hindering transcription, resulting in the apoptosis of cancer cells [77,78], and a higher induction of cytotoxicity. It has been reported that DOX does not assemble in the cytoplasm of cancer cells, due to the high P-glycoprotein (P-gp) expression on the cell membranes [79]. The DOX-CHI-MgFe_2_O^4^ complexes were the most effective in the cancer cells at 40 µg/mL. This can be attributed to the different internalization mechanisms of free DOX and DOX-CHI-MgFe_2_O_4_ MNPs. The DOX-CHI-MgFe_2_O_4_ complexes are readily taken up into the cells by endocytosis, but the MNP encapsulated DOX renders the P-gp incapable of pumping out the drug causing accumulation of DOX in the cells. Hence, the DOX-CHI-MgFe_2_O_4_ possessed higher cytotoxicity than the free DOX, which merely passes through the cell membrane [80].

The DOX-loaded MNPs also displayed greater anticancer activity in the SKBR-3 cells, resulting from the SKBR-3 cells possessing a lower P-gp expression on the cell membrane relative to the Caco-2 cells [81,82]. Although the cytotoxicity profiles of the DOX-loaded MNPs are similar, the higher cytotoxicity found in the SKBR-3 cells could be due to drug specificity at the tumor site [83] or possibly some cell specificity as well. With this in mind, the use of these MNPs as drug delivery vehicles in the treatment of breast cancer may be a possibility, and need to be explored further. The implementation of MNPs for the delivery of anticancer agents can provide several advantages relative to the free drug. The versatility of these ferrite MNPs provided an increase in the biodistribution of DOX, enabling the administration of higher doses of the chemotherapeutic drug [84].

From the observations of the apoptosis assay, it can be inferred that the DOX-loaded MNPs induced the formation of more apoptotic and necrotic bodies (Figure 8) and had higher apoptotic indices (Table 6) compared to that of the free DOX. The apoptotic indices for the Caco-2 and SKBR-3 cells were higher than that for the non-cancer HEK293 cells. This coincides with the observations from the drug release studies, where at physiological pH, there was a slow release of the bioactive drug. These results further validate the MTT cytotoxicity assay, thereby confirming the efficiency of these DOX-loaded MNPs, especially the DOX-CHI-MgFe_2_O_4_ MNPs in drug delivery.

## 4. Materials and Methods

### 4.1. Materials

Acridine orange hemi (zinc chloride) salt [3,6-Bis(dimethylamino) acridine hydrochloride zinc chloride double salt] (C_17_H_19_N_3_), chitosan (Shrimp Shells) (C_6_H_11_NO_4_, ≥75% deacetylated), ethanol (C_2_H_5_OH), 99.8%, ethylene glycol (CH_2_OH), iron (III) chloride tetrahydrate (FeCl_2_.4H_2_O), magnesium chloride hexahydrate (Cl_2_Mg.6H_2_O), poly(vinyl) alcohol (CH_2_CHOH_n_), dialysis tubing (MWCO = 14,000 Da) and doxorubicin hydrochloride (C_27_H_29_NO_11_.HCl) were purchased from Sigma-Aldrich (St. Louis, MO, USA). Silver nitrate (AgNO_3_), ethidium bromide (C_11_H_20_BrN_3_ ≥ 98%), polyethylene glycol 2000 [(HO(C_2_H_4_O)_n_H)], sodium chloride (NaCl), sodium hydroxide (NaOH), trichloroacetic acid (C_2_HCl_3_O_2_), 3-(4,5-Dimethylthiazol-2-yl)-2,5-diphenyltetrazolium bromide (MTT), HEPES buffered saline (HBS), dimethyl sulfoxide (DMSO), disodium phosphate (Na_2_HPO_4_), monopotassium phosphate (KH_2_PO_4_, potassium chloride (KCl) and Tris (hydroxymethyl)-aminomethane (Tris base solution) (C_4_H_11_NO_3_) were sourced from Merck (Darmstadt, Germany). Acetic acid (CH_3_COOH) was sourced from BDH Chemicals Ltd. (Poole, England). The human embryonic kidney (HEK293), colorectal adenocarcinoma (Caco-2), and breast adenocarcinoma (SKBR-3) cell lines were originally acquired from the American Type Culture Collection (ATCC, Manassas, VA, USA). Foetal bovine serum (FBS) was obtained from Hyclone GE Healthcare (South Logan, UT, USA). Eagle’s Minimum Essential Medium (EMEM), penicillin-streptomycin (100 U/mL penicillin, 100 µg/mL streptomycin) and trypsin were sourced from Lonza BioWhittaker (Verviers, Belgium). All other chemicals were sourced locally, with 18 MΩ (Milli-Q) water being used throughout.

### 4.2. Synthesis of MgFe_2_O_4_ Magnetic Nanoparticles (MNPs)

The MgFe_2_O_4_ MNPs were synthesized using the glycol-thermal method [85]. Approximately 3.4454 g of Cl_2_Mg.6H_2_O was dissolved in 500 mL of deionized water and stirred for 30 min. To ensure precipitation of the metal chlorides, a 5 M NaOH solution was added to the mixture until pH 9 was attained. The precipitate was washed with water to remove excess chlorides. The washed precipitate was then immersed into a 250 mL ethylene glycol solution and positioned for 6 h in a PARR 4843 stirred pressure reactor (PARR Instruments, Moline, IL, USA) at 300 rpm and 80 psi pressure, and a soak temperature of 200 °C. The final precipitate was washed with 200 mL of ethanol and positioned under a 200 W infrared light to dry overnight. An agate mortar and pestle were then used to homogenize the dried samples.

### 4.3. Functionalization of the MgFe_2_O_4_ MNPs with Chitosan (CHI)

CHI functionalized MNPs were synthesized with modifications from those described previously [12,21,86]. Approximately 0.5 g of the MgFe_2_O_4_ ferrite MNPs was added to a 0.5% CHI solution at pH 4.8, sonicated at 60 °C for 1 h using a Scientech Ultrasonic bath (Science Enterprises, New Delhi, India) followed by mechanical stirring using an IKA RW 20 Digital Dual-Range Mixer System (Cole-Parmer, Vernon Hills, IL, USA) for 18 h at room temperature. The black homogenous mixture (5% CHI functionalized MNPs) obtained was separated using an external magnetic field and dried overnight at room temperature.

### 4.4. Functionalization of the MgFe_2_O_4_ MNPs with Polyvinyl Alcohol (PVA)

PVA functionalization of the MgFe_2_O_4_ MNPs was adapted with modifications from previous reports [21,28]. Approximately 1 g of MgFe_2_O_4_ and 3 g PVA were added to 96 mL of Milli-Q water and stirred vigorously until a temperature of 80 °C was attained. This ensured complete dispersion of the hydrophilic polymer. The resulting solution was then stirred overnight at room temperature. The final 3% PVA functionalized MgFe_2_O_4_ MNPs were separated as in 4.2, rinsed five times with Milli-Q water to remove residual solvents and dried overnight at 35 °C.

### 4.5. Functionalization of the MgFe_2_O_4_ MNPs with Polyethylene Glycol (PEG)

PEG functionalized MgFe_2_O_4_ MNPs were prepared with modifications to previous reports [21,33]. Approximately 1 g of MgFe_2_O_4_ MNPs was added to 100 mL of Milli-Q water and sonicated for 30 min. A PEG_2000_ solution (3 g PEG_2000_ in 100 mL of deionized water) was then introduced into the homogenous MgFe_2_O_4_ solution and stirred overnight at room temperature. Thereafter, the PEG functionalized MNPs were rinsed five times with Milli-Q water to remove excess PEG_2000_ and dried overnight at 60 °C. The final 3% PEG functionalized MgFe_2_O_4_ MNPs were separated using an external magnet as in 4.2.

### 4.6. Fourier-Transform Infrared Spectroscopy (FTIR), X-ray Diffraction (XRD), and Vibrating Sample Magnetometry (VSM)

FTIR analysis was conducted in a Perkin Elmer Spectrum 100 FTIR spectrometer fitted with a Universal Attenuated Total Reflectance (ATR) component and Spectrum^®^ Software (PerkinElmer, Waltham, MA, USA). The FTIR spectral data for the MNPs, DOX and DOX-loaded MNPs were obtained in a wavelength range of 400–4000 cm^−1^.

XRD analysis was used to differentiate between the average crystalline sizes and the crystallinity of the ferrite MNPs. The XRD patterns of the MNPs were recorded with an Empyrean PANalytical X-ray diffractometer (Malvern Panalytical, Worcestershire, UK) with a monochromatic CoKα (1.788 Å) radiation at ambient temperature (10–80 °C) in a scale of 2θ. A step time of 3 s with a scanning speed of 0.002°/s was implemented. The analyses and indexing of the diffraction peaks were attained via the international centre of diffraction data (ICDD) database. The average crystalline sizes of the MNPs were acquired by applying Scherrer’s Equation (1) to the full width at half maximum (FWHM) of the most intense diffraction peak.

(1) $$D_{N}=\frac{k\lambda }{\beta \cos\cos\theta }$$

*D_N_* denotes the average crystalline size of the MNP, λ represents the wavelength of radiation, *k* symbolizes the shape function (0.9 is applied as a standard), and *β* signifies the FWHM which is measured in radians using the 2θ scale: where *θ* represents the Bragg angle.

Magnetic measurements of the MNPs were obtained using a LakeShore Model 735 Vibrating Sample Magnetometer (Lake Shore Cryotronics, Westerville, OH, USA), subjected to an applied magnetic field of 14 kOe at ambient temperature. The desired data was obtained by an inbuilt data acquisition software and interface card.

### 4.7. Encapsulation of Doxorubicin (DOX)

The encapsulation of DOX was adapted from that previously reported with modifications [87]. Approximately 5 mg MNPs were added to 12.5 mL of PBS (pH 7.4). The mixtures were gently stirred at 37 °C, followed by the addition of 2 mg of DOX to each MNP suspension. The resulting mixtures were then placed on an Infors HT Ecotron Shaking Incubator (United Scientific, Cape Town, South Africa) at 200 rpm for 48 h at ambient temperature. The DOX encapsulated MNPs were separated from the suspension using an external magnet. The samples were then washed (5×) to remove the residual unbound drug and dried overnight at room temperature.

The quantification of DOX in the DOX-loaded MNPs was determined using a Jasco V-730 Bio Spectrophotometer (JASCO Corporation, Hachioji City, Japan) at a wavelength of 481 nm. This was achieved by measuring the variance in the intensity between the total amount of DOX added and the amount of DOX present in the PBS. The encapsulation efficiency was calculated using Equation (2):

(2) $$\mathrm{Encapsulation}\;\mathrm{Efficiency}\;(\%)=\frac{\left(\mathrm{Total}\;\mathrm{DOX}\;\mathrm{added})-\;(\mathrm{free}\;\mathrm{DOX}\right)}{\left(\mathrm{Total}\;\mathrm{DOX}\;\mathrm{added}\right)}\;\times \;100$$

### 4.8. Transmission Electron Microscopy (TEM), Nanoparticle Tracking Analysis (NTA)

The size distribution, shape and morphological properties of the MNPs and DOX-loaded MNPs were determined using TEM. The MNP and DOX-loaded MNP samples were placed onto carbon-copper grids at ambient temperature and viewed using a JEM-1010 Transmission Electron Microscope (Jeol JEM 1010, Tokyo, Japan) functioning at an accelerated voltage of 100 kV. Micrographs were documented by the MegaView Ⅲ Soft Imaging Systems (SIS) side-mounted three-megapixel digital camera. Selection and visualization of preferred images were accomplished by using the associated SIS iTEM software. EDX and mapping analyses of the MNP samples were captured using the AZtecOne Software conjoined to an Oxford X-Max EDX Detector (Oxford Instruments, UK) at an accelerated voltage of 20 kV.

Stability, zeta potential and hydrodynamic sizes of the MNPs and DOX-loaded MNPs were obtained by NTA. Samples were diluted in ultrapure water (1:100), and the analyses were performed in a NanoSight NS500 (Malvern Instruments, Worcestershire, UK) at 25 °C. Data was analyzed using the associated NanoSight NTA 3.2 software. NTA was further employed to determine the physical interaction and stability of the respective nanoparticles. The method was adapted and modified from that recently reported in literature [59]. Nanocomplex sizes and zeta potential were determined before and after subjecting the respective nanocomplex to various conditions. Firstly, the nanocomplexes were sonicated for 30 s at 25 °C and at 4 °C. Secondly, the nanocomplexes were directly suspended in PBS at pH 4.5, 6.5 and 7.4 for 15 min before analysis to determine if there was any loss of integrity at high or low pH which could result in the drug leaching from the nanocomplex.

### 4.9. In Vitro Drug Release

Drug release studies were conducted to assess the ability of the nanocomplexes to release DOX over a duration of 72 h at physiological pH 7.4 and acidic pH of 6.5 and 4.5. Approximately 1.5 mg of the DOX-loaded MNP samples were placed and sealed in separate dialysis tubes (MWCO 12 000 Da) and dialyzed against PBS (5 mL) at 37 °C. At selected time intervals (0, 4, 8, 12, 16, 20, 24, 28, 32, 36, 40, 44, 48, 60 and 72 h), a 10 µL sample was removed and analyzed using a spectrophotometer at a wavelength of 481 nm. A subsequent 10 µL of PBS was replaced to maintain the sink volume.

### 4.10. MTT Cytotoxicity Assay

The cytotoxicity of the MNPs (10–100 µg/mL), DOX-loaded MNP formulations (4–40 µg/mL) and free DOX (4–40 µg/mL) were assessed using the MTT assay in the HEK293, Caco-2 and SKBR 3 cell lines. Upon confluency, the cells were trypsinized and seeded into 96-well plates at densities of 2 × 10^5^ cells per well and incubated at 37 °C overnight. Thereafter, the medium was removed, and the cells were supplemented with 100 µL of fresh complete medium (EMEM+ 10% FBS+ 1% antibiotics).

Cells were treated with various concentrations of MNPs, DOX and DOX-loaded MNPs in triplicate. Untreated cells were used as a positive control (100% cell viability). Following a 48-h incubation period at 37 °C, the spent medium was replaced with 100 µL of medium containing 10 µL of MTT solution (5 mg/mL in PBS). The cells were then incubated for 4 h at 37 °C. Thereafter, the medium/MTT mixture was removed, and 100 µL of DMSO was added to solubilize the formazan crystals, and absorbances measured at 570 nm in a MR-96A Microplate Reader (Vacutec, Hamburg, Germany). A background reading at 630 nm for nonspecific signals was measured and subsequently subtracted from the absorbance for the treated cells [88,89]. The cell viability (%) was calculated using Equation (3):

Cell viability (%) = (Abs of treated/Abs of control) × 100% (3)

### 4.11. Fluorescent Apoptosis Assay

The dual acridine orange/ethidium bromide (AO/EB) fluorescent staining can be employed to distinguish apoptotic related changes to the cell membrane during the progression of apoptosis [90,91]. Cells were seeded as in 4.9 and incubated overnight at 37 °C. The medium was then replenished, and the DOX-loaded MNPs were added using the half-maximal inhibitory concentrations (IC_50_) of the drug-loaded MNPs (Table 5) obtained from the MTT assay. The cells were incubated overnight at 37 °C, followed by removing the medium and washing the cells with 100 µL of PBS. After that, 10 µL of AO/EB dye was added, and the cells were stained for 5 min. The dye was then removed, and the cells were washed with PBS (100 µL), and viewed under an Olympus CKX41 inverted fluorescence microscope at 100X magnification, and images captured using a CC12 fluorescence camera (Olympus Co., Tokyo, Japan). Apoptotic indices were evaluated using Equation (4):

(4) $$\mathrm{Apoptotic}\;\mathrm{Index}=\frac{\mathrm{Number}\;\mathrm{of}\;\mathrm{apoptotic}\;\mathrm{cells}}{\mathrm{Total}\;\mathrm{number}\;\mathrm{of}\;\mathrm{cells}}$$

### 4.12. Statistical Analysis

All data are presented as mean ± standard deviation (±SD *n* = 3). Statistical analyses among mean values were performed using two-way ANOVA Turkey’s post hoc test. Statistical significance was set at * *p* < 0.05 and ** *p* < 0.01. Comparisons were conducted between the experimental data and their respective controls.

## 5. Conclusions

We have successfully shown the favorable therapeutic potential of these polymer functionalized MNPs for DOX delivery. The high DOX encapsulation and pH-responsive DOX release bode well for the use of these MNPs in nanomedicine. All DOX-loaded MNP formulations exhibited significant toxicity in the cancer cells, with a greater specificity towards the breast cancer cells (SKBR-3), implying a potential in breast cancer therapy. More specifically, the CHI-MgFe_2_O_4_ MNPs demonstrated the highest DOX encapsulation with over 80% of the drug released at a lower pH, a typical environment in cancer cells. This was closely followed by the PEG-MgFe_2_O_4_ and the PVA-MgFe_2_O_4_ MNPs, with the latter having a much lower DOX encapsulation and a burst release of DOX before a more sustained release was achieved. Overall, these results are encouraging and warrant further investigation and optimization before using these polymerized MNPS in vivo.

## Figures and Tables

**Table 1:** Structural parameters of the MgFe_2_O_4_, CHI-MgFe_2_O_4_, PVA-MgFe_2_O_4_ and PEG-MgFe_2_O_4_ ferrite NPs obtained from XRD measurements.

Ferrite NPs	Crystalline Size (*D*) (nm)	Lattice Parameter (Å)	Lattice Strain
MgFe_2_O_4_	18.38	8.348	0.0071
CHI-MgFe_2_O_4_	20.75	8.332	0.0063
PVA-MgFe_2_O_4_	19.86	8.330	0.0069
PEG-MgFe_2_O_4_	24.44	8.346	0.0059

**Table 2:** Magnetization measurements obtained for the MNPs.

Figure	Coercivity (*H_C_*) (KOe)	Saturation Magnetization (*M_S_*) (emu/g)
MgFe_2_O_4_	3.24	55.900
CHI-MgFe_2_O_4_	8.48	24.877
PVA-MgFe_2_O_4_	3.58	52.408
PEG-MgFe_2_O_4_	3.89	53.913

**Table 3:** Elemental composition of the (a) MgFe_2_O_4_, (b) CHI-MgFe_2_O_4_, (c) PVA-MgFe_2_O_4_ and (d) PEG-MgFe_2_O_4_ ferrite MNPs obtained from EDX (Figure 4B).

Element	(Wt%)			
	(a) MgFe_2_O_4_	(b) CHI-MgFe_2_O_4_	(c) PVA-MgFe_2_O_4_	(d) PEG-MgFe_2_O_4_
C	9.11	21.23	8.26	8.37
O	39.10	51.92	34.89	30.89
Mg	3.72	2.33	2.41	2.39
Fe	48.07	24.52	54.44	58.35

**Table 4:** Sizing and zeta potentials obtained for the MNPs and DOX-loaded MNPs.

MNPs/DOX-MNPs	TEM Particle Size (nm)	Hydrodynamic Size (Mean ± Standard Error)	Zeta Potential (Mean ± Standard Error)	Polydispersity Index (PDI)
MgFe_2_O_4_	18.38 ± 1.3	91.5 ± 15.3 nm	−6.3 ± 1.2 mV	0.028
CHI-MgFe_2_O_4_	21.00 ± 0.9	116.7 ± 18.3 nm	−11.5 ± 0.3 mV	0.025
PVA-MgFe_2_O_4_	19.15 ± 1.2	99.7 ± 4.9 nm	−57.0 ± 0.0 mV	0.0024
PEG-MgFe_2_O_4_	23.28 ± 2.1	139.4 ± 21.0 nm	−27.1 ± 3.6 mV	0.023
DOX-CHI-MgFe_2_O_4_	16.24 ± 0.7	78.9 ± 4.5 nm	−21.8 ± 0.2 mV	0.0033
DOX-PVA-MgFe_2_O_4_	17.65 ± 0.5	87.2 ± 11.3 nm	−25.2 ± 0.4 mV	0.017
DOX-PEG-MgFe_2_O_4_	20.86 ± 1.3	98.8 ± 4.3 nm	−27.3 ± 3.6 mV	0.0019

**Table 5:** IC_50_ (µg) values for DOX-loaded MNPs and free DOX.

	Drug Nanocomplexes			
Cells	DOX	DOX-CHI-MgFe_2_O_4_	DOX-PVA-MgFe_2_O_4_	DOX-PEG-MgFe_2_O_4_
HEK293	39.98 ± 0.3	18.2 ± 0.5	5.6 ± 0.1	125.9 ± 1.2
Caco-2	67.61 ± 1.1	11.75 ± 0.3	12.3 ± 0.2	7.94 ± 0.5
SKBR-3	15.85 ± 0.4	9.12 ± 0.3	3.63 ± 0.06	14.13 ± 0.9

**Table 6:** Pseudo IC_50_ (µg) values for DOX-loaded MNPs.

Drug Nanocomplexes			
Cells	DOX-CHI-MgFe_2_O_4_	DOX-PVA-MgFe_2_O_4_	DOX-PEG-MgFe_2_O_4_
HEK293	18.2 ± 0.3	2.95 ± 0.07	100.64 ± 1.1
Caco-2	9.77 ± 0.1	6.31 ± 0.09	6.28 ± 0.08
SKBR-3	7.59 ± 0.07	1.897 ± 0.01	11.22 ± 0.9

**Table 7:** Apoptotic Indices for DOX-loaded MNPS and free DOX.

	Drug Nanocomplexes			
Cells	DOX	DOX-CHI-MgFe_2_O_4_	DOX-PVA-MgFe_2_O_4_	DOX-PEG-MgFe_2_O_4_
HEK293	0.38 ± 0.004	0.44 ± 0.002	0.41 ± 0.003	0.47 ± 0.001
Caco-2	0.4 ± 0.001	0.46 ± 0.002	0.5 ± 0.004	0.7 ± 0.012
SKBR-3	0.47 ± 0.003	0.5 ± 0.004	0.58 ± 0.004	0.69 ± 0.010

## Data Availability

The data and contributions presented in the study are included in the article. Further inquiries can be directed to the corresponding author.

## Supplementary Materials

The following are available online, Figure S1: FTIR spectra of (a) polyvinyl alcohol, (b) chitosan and (c) polyethylene glycol. Table S1. Effect of pH and temperature on the size and stability of the DOX-CHI-MgFe_2_O_4_ nanocomplexes. Table S2. Effect of pH and temperature on the size and stability of the DOX-PVA-MgFe_2_O_4_ nanocomplexes. Table S3. Effect of pH and temperature on the size and stability of the DOX-PEG-MgFe_2_O_4_ nanocomplexes.
