# Supplementary material for: Chitosan, Polyethylene Glycol and Polyvinyl Alcohol Modified MgFe2O4 Ferrite Magnetic Nanoparticles in Doxorubicin Delivery: A Comparative Study In Vitro
Source: Molecules. 2021 Jun 25;26(13):3893. doi: 10.3390/molecules26133893 (PMC8271878; doi:10.3390/molecules26133893)
Supplement: Supplementary file 1 [file molecules-26-03893-s001.zip › molecules-1244398-supplementary.pdf]

molecules

Supplementary Material

Chitosan, Polyethylene glycol and Polyvinyl Alcohol Modified $MgFe_2O_4$ Ferrite Magnetic Nanoparticles in Doxorubicin Delivery: A comparative Study In Vitro

Deevak Ramnandan, Seipati Mokhosi, Aliscia Daniels and Moganavelli Singh *

Nano-Gene and Drug Delivery Group, Discipline of Biochemistry, University of KwaZulu-Natal, Private Bag X54001, Durban, South Africa; deevaksyn@gmail.com (D.R.); MOKHOSIS@ukzn.ac.za (S.M.); DanielsA@ukzn.ac.za (A.D.)

* Correspondence: singhm1@ukzn.ac.za; Tel.: +27-31-2607170

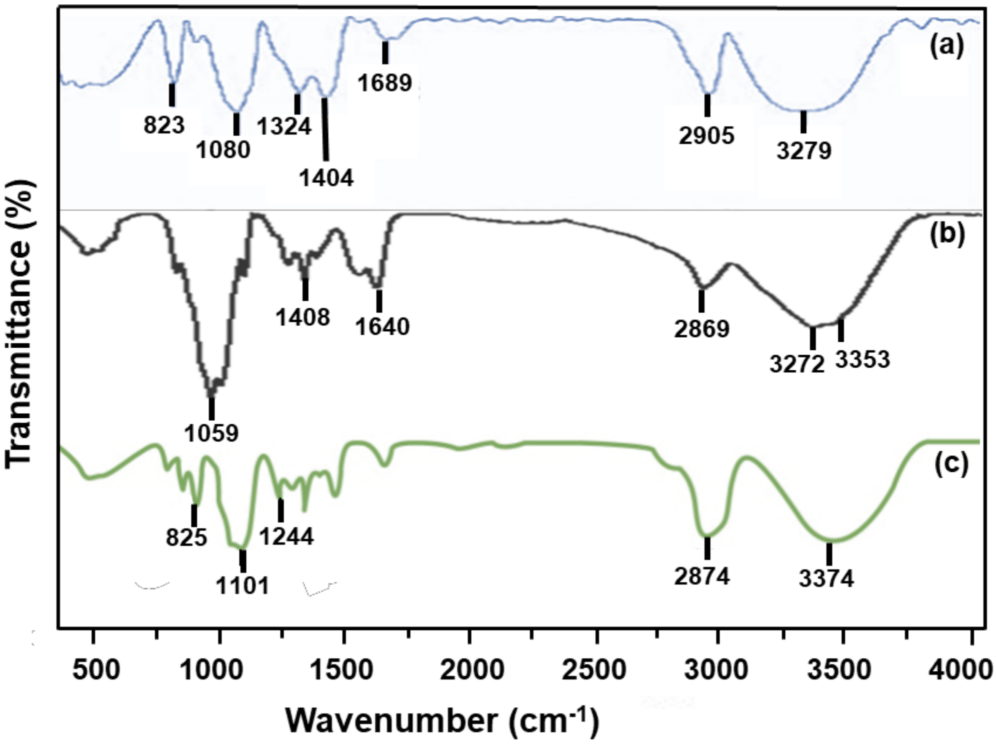

Figure S1. FTIR spectra of (a) Polyvinyl alcohol, (b) Chitosan and (c) Polyethylene glycol

Table S1. Effect of pH and temperature on the size and stability of the DOX-CHI-$MgFe_2O_4$ nanocomplexes.

	Size (nm)	Zeta Potential (mV)
**pH 4.5**	120.9 ± 5.9	-5.1 ± 0.8
**pH 6.5**	80.3 ± 2.8	-13.1 ± 1.5
**pH 7.4**	79.9 ± 18.1	-13.5 ± 2.5
**4 ˚C**	85.6 ± 10.4	-9.4 ± 1.1
**25 ˚C**	80.1 ± 7.5	14.7 ± 0.5

Table S2. Effect of pH and temperature on the size and stability of the DOX-PVA-$MgFe_2O_4$ nanocomplexes.

	Size (nm)	Zeta Potential (mV)
**pH 4.5**	135.6 ± 20.8	-11.5 ± 6.8
**pH 6.5**	111.3 ± 15.7	-20.4 ± 3.7
**pH 7.4**	98.4 ± 34.8	-11.2 ± 0.1
**4 ˚C**	62.0 ± 1.2	- 14.5 ± 2.8
**25 ˚C**	77.0 ± 29.7	- 10.6 ± 0.2

Table S3. Effect of pH and temperature on the size and stability of the DOX-PEG-$MgFe_2O_4$ nanocomplexes.

	Size (nm)	Zeta Potential (mV)
**pH 4.5**	104.6 ± 19.2	4.4 ± 0.4
**pH 6.5**	95.3 ± 9.3	- 14.7 ± 0.2
**pH 7.4**	80.4 ± 4.6	-15.3 ± 5.4
**4 ˚C**	123.8 ± 20.7	-4.4 ± 1.4
**25 ˚C**	82.6 ± 9.2	- 12.6 ± 4.7
